# Therapeutic effects of eperisone on pulmonary fibrosis via preferential suppression of fibroblast activity

**DOI:** 10.1038/s41420-022-00851-7

**Published:** 2022-02-08

**Authors:** Ken-ichiro Tanaka, Mikako Shimoda, Toshifumi Sugizaki, Maki Ikeda, Ayaka Takafuji, Masahiro Kawahara, Naoki Yamakawa, Tohru Mizushima

**Affiliations:** 1 Laboratory of Bio-Analytical Chemistry, Research Institute of Pharmaceutical Sciences, Faculty of Pharmacy, Musashino University, 1-1-20 Shinmachi, Nishi-Tokyo, 202-8585 Japan; 2 Shujitsu University School of Pharmacy, Okayama, 703-8516 Japan; 3 LTT Bio-Pharma Co., Ltd, Shiodome Building 3F, 1-2-20 Kaigan, Minato-ku, Tokyo 105-0022 Japan

**Keywords:** Experimental models of disease, Respiratory tract diseases, Drug development

## Abstract

Although the exact pathogenesis of idiopathic pulmonary fibrosis (IPF) is still unknown, the transdifferentiation of fibroblasts into myofibroblasts and the production of extracellular matrix components such as collagen, triggered by alveolar epithelial cell injury, are important mechanisms of IPF development. In the lungs of IPF patients, apoptosis is less likely to be induced in fibroblasts than in alveolar epithelial cells, and this process is involved in the pathogenesis of IPF. We used a library containing approved drugs to screen for drugs that preferentially reduce cell viability in LL29 cells (lung fibroblasts from an IPF patient) compared with A549 cells (human alveolar epithelial cell line). After screening, we selected eperisone, a central muscle relaxant used in clinical practice. Eperisone showed little toxicity in A549 cells and preferentially reduced the percentage of viable LL29 cells, while pirfenidone and nintedanib did not have this effect. Eperisone also significantly inhibited transforming growth factor-β1-dependent transdifferentiation of LL29 cells into myofibroblasts. In an in vivo study using ICR mice, eperisone inhibited bleomycin (BLM)-induced pulmonary fibrosis, respiratory dysfunction, and fibroblast activation. In contrast, pirfenidone and nintedanib were less effective than eperisone in inhibiting BLM-induced pulmonary fibrosis under this experimental condition. Finally, we showed that eperisone did not induce adverse effects in the liver and gastrointestinal tract in the BLM-induced pulmonary fibrosis model. Considering these results, we propose that eperisone may be safer and more therapeutically beneficial for IPF patients than current therapies.

## Introduction

Idiopathic pulmonary fibrosis (IPF) is a lung disease of unknown etiology with a poor prognosis that is characterized by chronic development of severe fibrosis, resulting in a honeycomb lung [1, 2]. Steroids and immunosuppressive drugs have long been used to treat IPF, but in many cases, these drugs do not show therapeutic efficacy against IPF progression [2–4]. The primary etiology of IPF is thought to be chronic pulmonary fibrosis triggered by chronic injury to airway and alveolar epithelial cells. Therefore, pirfenidone and nintedanib, antifibrotic agents that have been shown to significantly improve the reduction of forced vital capacity (FVC) in large clinical trials, are being used to treat IPF in clinical practice [2, 5, 6]. However, in some cases, these drugs have not shown efficacy and have been reported to induce adverse effects such as elevation of liver damage markers, diarrhea, and indigestion [5, 6]. Thus, safer drugs eliciting a therapeutic effect equal to or greater than that of these two approved drugs are necessary.

Although the exact cause of IPF is unknown, it is thought to be triggered by damage to the lung epithelium as a result of increased oxidative stress and the repair and remodeling processes, such as collagen synthesis, that are induced to manage this damage. In other words, this process is overstimulated, resulting in abnormal wound repair and remodeling characterized by collagen deposition, which leads to the development and exacerbation of pulmonary fibrosis [7, 8]. The cells that play the greatest role in this fibrosis-promoting process are myofibroblasts. Peribronchial and perivascular fibroblasts transdifferentiate (activate) into myofibroblasts in response to various stimuli, especially TGF-β1, and accumulate extracellular matrix components, especially collagen fibers, which are involved in fibrosis [9, 10]. Furthermore, the “apoptosis paradox” is also a possible mechanism of abnormal fibrosis in IPF patients. Apoptosis is preferentially observed in alveolar epithelial cells in the lungs of IPF patients, while little apoptosis occurs in fibroblasts. This produces a relative imbalance resulting in increased fibroblasts in the lungs of IPF patients, which is thought to be involved in the pathogenesis of IPF [11, 12]. Thus, it is important to identify compounds that inhibit transdifferentiation of fibroblasts into myofibroblasts or inhibit activation of myofibroblasts. In addition, compounds that are not toxic to alveolar epithelial cells but exert their effects preferentially on lung fibroblasts are promising candidates for IPF therapy.

On the basis of these requirements, we implemented an innovative research strategy (drug repositioning) to identify and develop new IPF therapeutics by screening drugs currently in clinical use to treat other diseases [13, 14]. The major advantage of this strategy is that the clinical safety of the drugs screened is already understood, and the risk of unexpected adverse effects in humans can be greatly reduced when these drugs are applied to treat other diseases [13, 14]. Using this strategy, we screened drugs not only for IPF but also chronic obstructive pulmonary disease, functional dyspepsia, and irritable bowel syndrome from a library of approved drugs, identified effective drugs for each disease, and analyzed the mechanisms by which these drugs exert their efficacy [15–18]. Recently, other research groups have used this strategy to develop novel therapeutics for coronavirus infection 2019, and candidate drugs have been discovered, including the influenza virus treatment drug remdesivir and the antiparasitic drug ivermectin [19]. Therefore, we suggest that this strategy is useful for discovering new candidates for the treatment of human diseases.

In our previous study, we screened compounds capable of more potently inhibiting the growth of lung fibroblasts (LL29 cells) than that of lung alveolar epithelial cells (A549 cells) and identified idebenone, which has previously been used clinically as a brain metabolic stimulant, from a library of medications already in clinical use. In addition, intratracheal administration of idebenone to mice inhibited bleomycin (BLM)-induced pulmonary fibrosis and decreased FVC [15]. Furthermore, in our previous screening, we found that, in addition to idebenone, the central muscle relaxant eperisone also acts preferentially on lung fibroblasts. No studies have been conducted on eperisone to determine its effects on fibroblasts or pulmonary fibrosis. Therefore, in this study, we investigated the effect of eperisone, which preferentially induces fibroblast cell death, on BLM-induced pulmonary fibrosis. In addition, we examined its adverse effects by analyzing plasma markers and the gastrointestinal mucosal status when eperisone was administered to BLM-induced pulmonary fibrosis model mice.

## Materials and methods

### Animals

ICR mice (6-7 weeks old, male) were purchased from Charles River (Yokohama, Japan). The experiments and procedures described here were carried out in accordance with the Guide for the Care and Use of Laboratory Animals as adopted and promulgated by the National Institutes of Health, and were approved by the Animal Care Committee of Musashino University.

### Treatment of mice with BLM, eperisone, and other reagents

Mice were anesthetized with isoflurane and intratracheally administered BLM (1 mg/kg, once) in sterile saline via a single channel pipette (P200). Ten days after BLM administration, eperisone (15 or 50 mg/kg), tolperisone (15 mg/kg), pirfenidone (200 mg/kg), and nintedanib (30 mg/kg) were administered orally for a total of 9 days from day 10 to day 18. Various analyses were then performed on day 20.

In the adverse effect study, 10 days after BLM administration, 250 mg/kg of eperisone was orally administered once, which was five times the dose that showed efficacy. Twenty-four hours after eperisone administration, the fecal condition of the mice was visually examined. In addition, plasma samples and stomach and colon tissues were collected from the mice. Analysis of the plasma samples was performed by TRANS GENIC INC. (https://www.transgenic.co.jp/).

### Measurement of lung mechanics and FVC

Measurement of lung mechanics and FVC was performed with a computer-controlled small-animal ventilator connected to a negative pressure reservoir (FlexiVent; SCIREQ, Montreal, Canada), as previously described [20]. Total respiratory system elastance and tissue elastance were measured by snap shot and forced oscillation techniques, respectively. For determination of FVC, lungs were inflated to 30 cmH_2_O over one second and held at this pressure. After 0.2 s, the pinch valve (connected to the ventilator) was closed, and after 0.3 s, the shutter valve (connected to the negative pressure reservoir) was opened, exposing the lung to the negative pressure, which was held for 1.5 sec to ensure complete expiration. All data were analysed using FlexiVent software (version 5.3; SCIREQ, Montreal, Canada).

### Statistical analysis

All values are expressed as mean ± S.E.M. One-way ANOVA, followed by Dunnett’s test, or Student’s unpaired *t* test were used to evaluate differences between three or more groups or between two groups, respectively. Mac statistical analysis Ver.3.0 software (Esumi Co., Ltd., Tokyo, Japan) was used for the statistical analyses. Differences were considered to be significant when *P* < 0.05.

## Results

### Preferential suppression of fibroblast activity by eperisone

A library of drugs already in clinical use was screened to identify drugs that are not toxic to alveolar epithelial cells but are preferentially toxic to lung fibroblasts. Specifically, LL29 or A549 cells were treated with each drug, and 24 h later, the percentages of viable cells were determined using the methylthiazole tetrazolium reagent. Among the drugs that showed lower IC50 values in LL29 cells than in A549 cells, idebenone and eperisone were selected based on the difference in IC50 values between the two cell types, their clinical safety, and other pharmacological activities. As described above, we previously reported the preferential suppression of fibroblast activity by idebenone and its efficacy against BLM-induced pulmonary fibrosis [15]. Therefore, in this study, we focused on eperisone, which is used in clinical practice as a central muscle relaxant [21], and examined its efficacy against IPF using in vitro and in vivo systems.

**Fig. 1:**
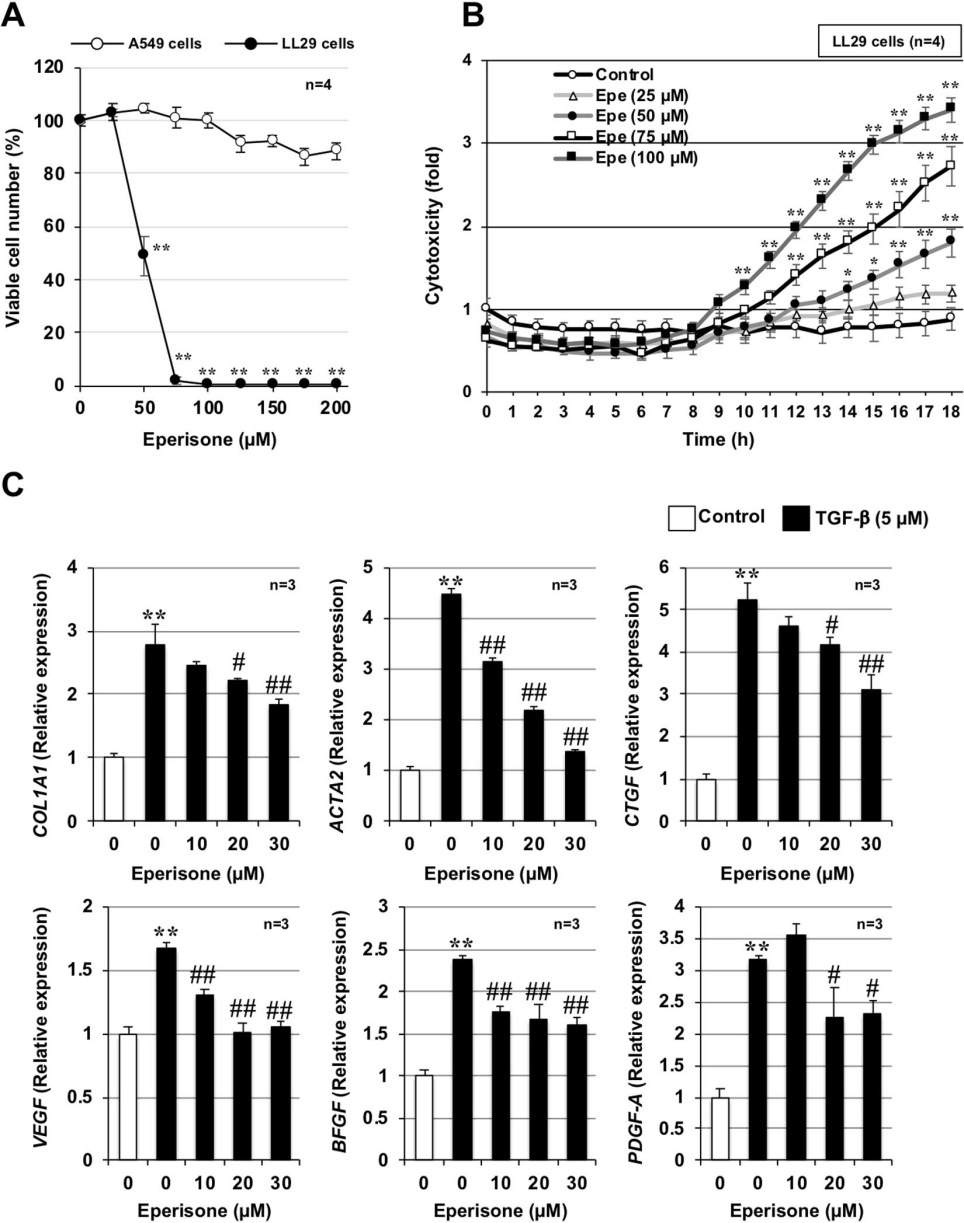
Preferential suppression of fibroblast activity by eperisone. LL29 or A549 cells were incubated with the indicated concentrations (µM) of eperisone for 24 h. The percentage of viable cells was determined using a CellTiter-Glo® 2.0 assay (**A**). LL29 cells were incubated with the indicated concentrations (µM) of eperisone for 18 h. The cytotoxicity was measured every hour using CellTox™ Green Dye and a microplate reader (excitation: 485 nm, emission: 530 nm) (**B**). LL29 cells were incubated with transforming growth factor (TGF)-β1 (5 ng/ml) for 72 h in the presence of the indicated concentrations of eperisone. Total RNA was extracted and subjected to real-time RT-PCR using a specific primer set for each gene. The values were normalized to *GAPDH* gene expression and expressed relative to the control sample (**C**). Values represent the mean ± SEM ** *or* ^*##*^*P* < 0.01; * or ^*#*^*P* < 0.05. (*, vs A549 cells (A) or Control (B, C); ^#^, vs TGF-ß (C)).

As shown in Fig. 1A, eperisone treatment (25–200 µM) decreased the percentage of viable LL29 cells in a dose-dependent manner. In contrast, the percentage of viable A549 cells treated with 200 µM of eperisone was 88.5 ± 3.0% (mean ± SEM, *n* = 4), revealing almost no decrease in viable A549 cells after eperisone treatment. In addition, eperisone was also found to reduce the number of viable cells in other fibroblasts (HFL-1 and IMR-90 cells) in a dose-dependent manner (Supplementary Fig. S1A). Furthermore, eperisone showed little toxicity to RL-34 cells (rat liver-derived normal epithelial cells) and preferentially decreased the number of viable cells in RI-T cells (rat hepatic stellate cells), which differentiate into myofibroblasts (Supplementary Fig. S1B). We next examined eperisone-induced cytotoxicity in LL29 cells using CellTox™ Green Dye, which can detect cell membrane disruption. As shown in Fig. 1B, LL29 cells treated with eperisone exhibited cytotoxic effects in a time- and concentration-dependent manner. Furthermore, we compared the effect of eperisone on TGF-β1–induced activation of lung fibroblasts. LL29 cells were pre-treated with eperisone (10–30 µM), followed by the addition of TGF-β1 (5 µM), and the expression of fibrosis-related factors was analyzed 72 h later by real-time RT-PCR. As shown in Fig. 1C, TGF-β1 increased the mRNA expression of Collagen 1a1 *(COL1A1)*, α-SMA *(ACTA2)*, connective tissue growth factor (*CTGF*), vascular endothelial growth factor (*VEGF*), basic fibroblast growth factor (*BFGF*), and platelet-derived growth factor (*PDGF-A*) in LL29 cells, but this increase was suppressed by pre-treatment with eperisone. These results suggest that eperisone preferentially suppressed lung fibroblast activity in vitro.

### Effects of other drugs on lung fibroblast viability

**Fig. 2:**
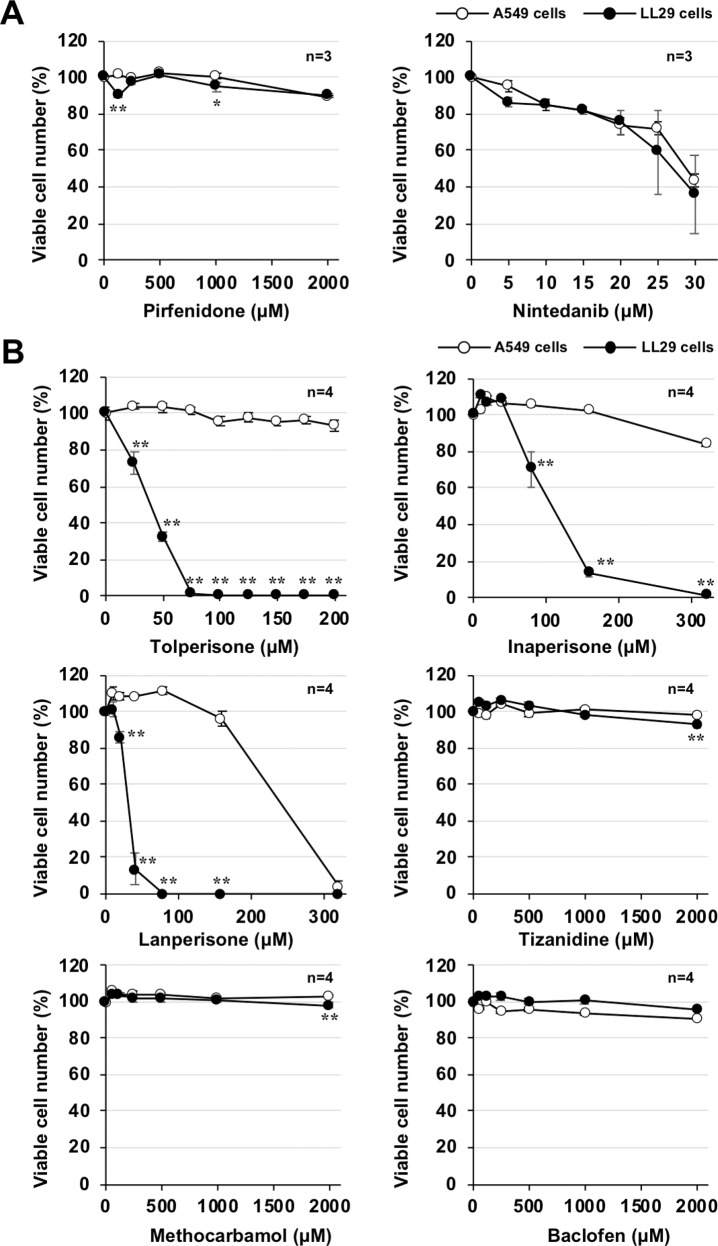
Effect of other drugs on the percentage of viable LL29 and A549 cells. LL29 or A549 cells were incubated with the indicated concentrations of pirfenidone, nintedanib (**A**), tolperisone, inaperisone, lanperisone, tizanidine, methocarbamol, or baclofen (**B**) for 24 h. The percentage of viable cells was determined using a CellTiter-Glo® 2.0 assay. Values represent the mean ± SEM **P < 0.01; *P < 0.05. (*, vs A549 cells).

As described in the introduction, pirfenidone, and nintedanib have been used as anti-fibrotic agents in clinical practice to treat IPF patients. Thus, to investigate the characteristic effect of eperisone on lung fibroblasts, we measured the percentages of viable LL29 and A549 cells after treatment with these existing drugs. After pirfenidone treatment (up to 2 mM), almost no decrease was observed in the percentage of viable cells of both cell types. In contrast, nintedanib decreased the percentage of viable cells of both cell types, but there was no difference in the degree of decrease between the cell types (Fig. 2A).

Eperisone is a central muscle relaxant that has been used in clinical practice to improve muscle tone in patients with lumbago and spastic paralysis caused by cerebrovascular disease. Thus, we determined whether other central muscle relaxants exert preferential effects on fibroblasts. Among the six drugs examined, tolperisone, inaperisone, and lanperisone preferentially reduced the viability of LL29 cells, similar to eperisone. However, tizanidine, methocarbamol, and baclofen, at concentrations up to 2 mM, did not reduce the viability of either cell type (Fig. 2B). As will be discussed in detail later, because preferential suppression of fibroblasts was not observed for some central muscle relaxants, we speculate that eperisone exerts its preferential effects by a molecular mechanism other than its muscle relaxant effect.

### Effect of eperisone on BLM-induced pulmonary fibrosis

**Fig. 3:**
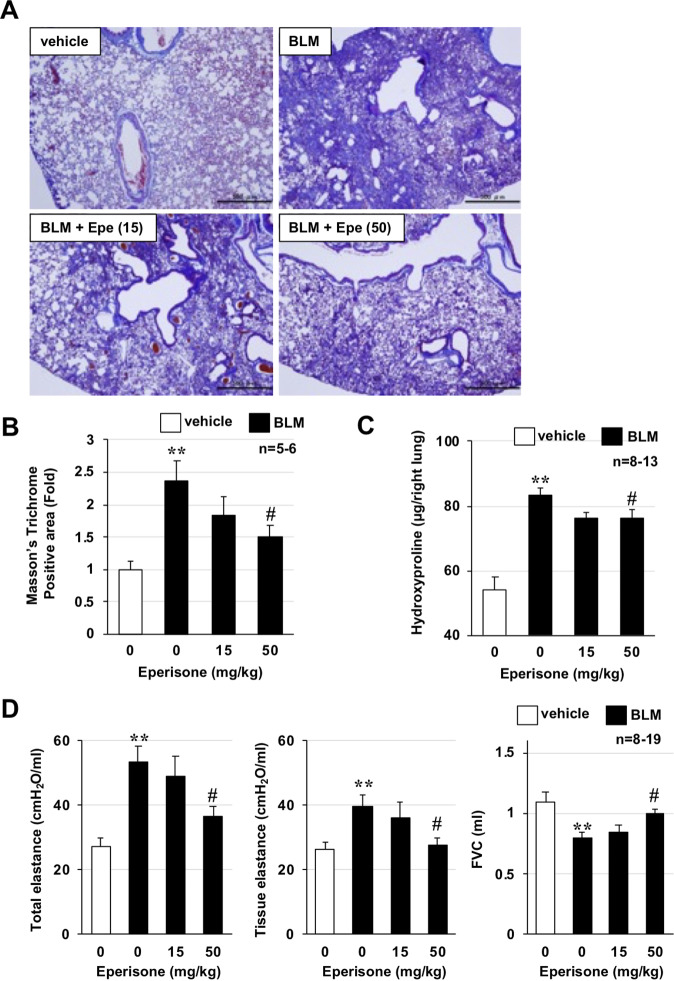
Effect of eperisone on pre-developed pulmonary fibrosis. Mice were treated with bleomycin (BLM, 1 mg/kg) or vehicle once only on day 0. The mice were then orally administered the indicated dose of eperisone (Epe) once daily for 9 days (from day 10 to day 18). Pulmonary tissue sections were prepared on day 20 and subjected to a histopathological examination (Masson’s trichrome staining; scale bar = 500 µm) (**A**). The collagen-positive area was determined based on Masson’s trichrome staining images (**B**). The pulmonary hydroxyproline level was determined on day 20 (**C**). The total respiratory system elastance, tissue elastance, and forced vital capacity (FVC) were measured on day 20 (**D**). Values represent the mean ± SEM **P < 0.01; ^#^*P* < 0.05; NS not significant. (*, vs vehicle; ^#^, vs BLM alone).

Pulmonary fibrosis was induced by intratracheal administration of BLM to male ICR mice. Specifically, 10 days after BLM administration, mice were divided into three groups based on the rate of change in body weight (excluding the vehicle group), and the effect of oral eperisone administration on lung fibrosis was examined. At 20 days after BLM administration, lung tissue sections were prepared and stained for collagen using Masson’s trichrome stain. Collagen deposition in the lungs was observed in a BLM administration-dependent manner. In contrast, oral eperisone administration suppressed the BLM-dependent collagen deposition in a dose-dependent manner (Fig. 3A, B). Next, we performed quantitative analysis of hydroxyproline, a collagen-specific amino acid, in lung tissue. As shown in Fig. 3C, BLM treatment significantly increased the amount of hydroxyproline in lung tissue, while eperisone treatment suppressed this increase. When considering the clinical application of eperisone for the treatment of lung fibrosis, it is important to improve respiratory function as well as histological and biochemical indices. Moreover, our previous analysis showed that lung elastance is increased and FVC is decreased in BLM-induced pulmonary fibrosis [15, 16]. Thus, we measured the respiratory function of mice using a computer-controlled ventilator and negative pressure reservoir. As shown in Fig. 3D, BLM treatment increased the total elastance (elastance of the entire lung including the bronchi, bronchioles, and alveoli) and tissue elastance (elastance of the alveoli) and decreased the FVC. In contrast, eperisone significantly improved the deterioration of respiratory function induced by BLM administration. These results indicate that eperisone has an ameliorating effect on BLM-dependent pulmonary fibrosis.

**Fig. 4:**
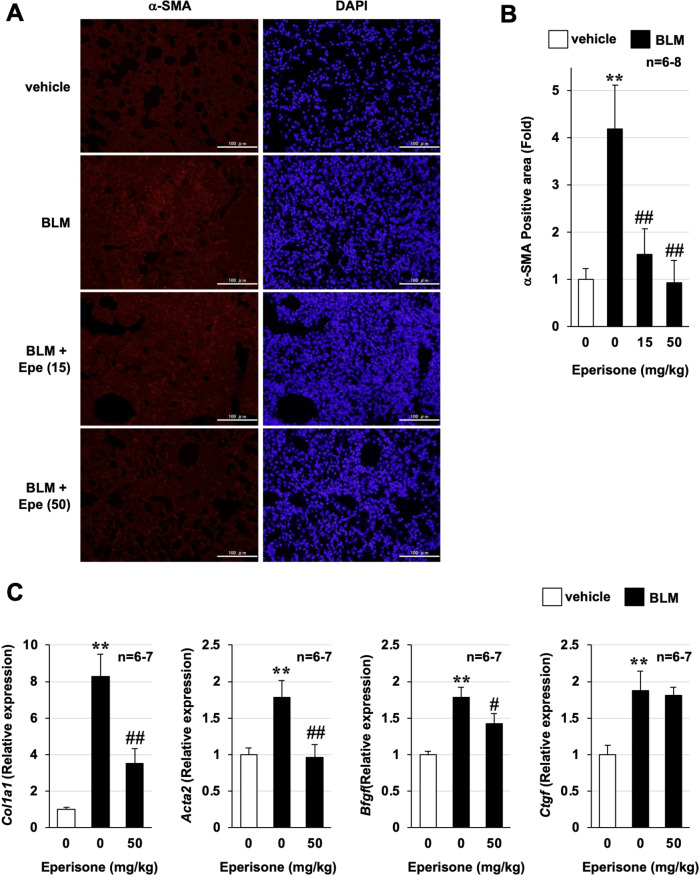
Effect of eperisone on bleomycin-induced increases in myofibroblasts. Mice were treated with bleomycin (BLM, 1 mg/kg) or vehicle once only on day 0. The mice were then orally administered the indicated dose of eperisone (Epe) once daily for 9 days (from day 10 to day 18). Pulmonary tissue sections were prepared on day 20 and subjected to immunohistochemical analysis with an antibody against α-smooth muscle actin (SMA) (scale bar = 100 µm) (**A**). The α-SMA-positive area was determined using ImageJ software (**B**). Total RNA was extracted from lung tissue and subjected to real-time RT-PCR using a specific primer set for each gene. The values were normalized to *Hprt1* gene expression and expressed relative to the control sample (**C**). Values represent the mean ± SEM ** or ^##^*P* < 0.01; ^*#*^*P* < 0.05. (*, vs vehicle; ^#^, vs BLM alone).

Fibroblasts are known to differentiate into myofibroblasts upon activation and play an important role in the development and exacerbation of lung fibrosis [9, 22]. Thus, we performed an immunohistochemical analysis of α-SMA, a myofibroblast marker. As shown in Fig. 4A, BLM treatment increased the number of α-SMA-positive cells in the lung, i.e., myofibroblasts increased in a BLM-dependent manner. In contrast, administration of 50 mg/kg eperisone decreased the BLM-dependent increase in α-SMA-positive cells in the lung to the same level as observed in the vehicle group. Moreover, as shown in Fig. 4C, BLM treatment increased the mRNA expression of *Col1a1*, *Acta2*, *Bfgf*, and *Ctgf* in the lungs of mice, while eperisone significantly suppressed the BLM-dependent increase of *Col1a1*, *Acta2*, and *Bfgf* expression. These results suggest that eperisone inhibits fibroblast activation not only in vitro but also in vivo and that it suppresses BLM-dependent pulmonary fibrosis through its inhibitory effect on fibroblast activation.

### Effects of other drugs on BLM-induced pulmonary fibrosis

Using the same experimental approach as in Fig. 3, we examined the effect of pirfenidone and nintedanib on BLM-dependent pulmonary fibrosis. The dose of pirfenidone (200 mg/kg) was 20 times the initial clinical dose (600 mg/day) used in Japan [23], and the same dose of nintedanib (30 mg/kg) that had shown triple kinase inhibition in previous animal studies was used [24]. As shown in Supplementary Fig. S2A–C, BLM-dependent collagen deposition and increased hydroxyproline levels in the lung were not suppressed by oral administration of pirfenidone or nintedanib. Nintedanib or pirfenidone administration tended to improve the BLM-dependent deterioration of respiratory function, but the effect was not statistically significant (Supplementary Fig. S2D).

We next analyzed the effect of tolperisone, a structural analogue of eperisone with central muscle relaxant properties [25], on BLM-dependent pulmonary fibrosis. As shown in Supplementary Fig. S3A–C, oral administration of tolperisone suppressed BLM-dependent collagen deposition and increased hydroxyproline levels in the lung similar to eperisone administration. In addition, tolperisone significantly improved the BLM-dependent increase in total elastance and tissue elastance and the decrease in FVC (Supplementary Fig. S3D). These results indicate that preferential suppression of fibroblast activity is important to prevent BLM-dependent exacerbation of lung fibrosis.

### Safety analysis of eperisone administration

**Fig. 5:**
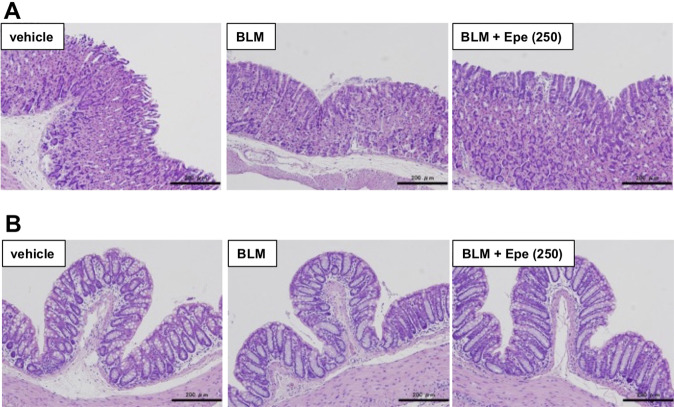
Effects of eperisone administration on gastric and colonic mucosa. Mice were treated with bleomycin (BLM, 1 mg/kg) or vehicle once only on day 0. The mice were then orally administered 250 mg/kg of eperisone (Epe) once at day 10. After 24 h, the stomach and colon were collected from the mice. Gastric (**A**) and colonic (**B**) tissue sections were prepared and subjected to histopathological examination (hematotoxin and eosin staining; scale bar = 200 µm).

In clinical practice, existing IPF treatments, such as pirfenidone and nintedanib, have been reported to induce adverse effects such as increasing markers of liver damage in the plasma and gastrointestinal disorders [5, 6]. Therefore, we conducted a comprehensive analysis of markers for pancreatic, hepatic, and renal damage in plasma. The dose of eperisone was five times higher than the dose that showed efficacy for BLM-dependent pulmonary fibrosis. As shown in Table 1, administration of BLM or BLM plus eperisone (250 mg/kg, once at day 10) did not significantly alter 12 plasma markers for pancreatic, hepatic, and renal damage. Moreover, administration of eperisone (50 mg/kg) for 9 consecutive days (from day 10 to day 18) did not cause any significant changes in the four plasma markers indicating hepatic and renal damage (Supplementary Table S1). In addition, no mouse exhibited diarrhea or hemorrhagic stool in either group (Table 2). Furthermore, we also examined gastric and colonic mucosal injury using hematoxylin and eosin staining. As shown in Fig. 5 and Table 2, the condition of the gastric and colonic mucosa in mice treated with BLM or BLM plus eperisone (250 mg/kg) was unchanged compared with that in vehicle-treated mice, and no gastric and colonic mucosal injury was observed. These results suggest that eperisone may be able to suppress pulmonary fibrosis without inducing adverse effects.

**Table 1:** Effect of eperisone administration on biochemical markers in the blood.

	vehicle (*n* = 4)	BLM (*n* = 4)	BLM + Epe (250) (*n* = 4)
Total protein (g/dL)	5.28 ± 0.40	4.70 ± 0.45	5.65 ± 0.17
Albumin (g/dL)	2.48 ± 0.10	2.28 ± 0.14	2.55 ± 0.03
A/G ratio	0.90 ± 0.04	0.98 ± 0.09	0.83 ± 0.03
Total bilirubin (mg/dL)	>0.1	>0.1	>0.1
AST (U/L)	51.5 ± 7.1	50.5 ± 6.4	55.8 ± 5.0
ALT (U/L)	20.0 ± 1.6	22.8 ± 4.8	22.8 ± 2.5
ALP (U/L)	126.8 ± 14.1	124.3 ± 14.4	98.5 ± 15.6
Amylase (U/L)	2469.5 ± 14.1	2166.8 ± 160.6	2549.3 ± 44.4
BUN (mg/dL)	16.3 ± 0.27	11.3 ± 0.94	13.9 ± 1.13
Creatinine (mg/dL)	>0.1	>0.1	>0.1
Total cholesterol (mg/dL)	119.3 ± 10.5	94.8 ± 7.9	113.3 ± 7.8
Blood glucose (mg/dL)	265.3 ± 50.3	227.3 ± 30.9	317.0 ± 32.4

Mice were treated with bleomycin (BLM, 1 mg/kg) or vehicle once only on day 0. The mice were then orally administered 250 mg/kg of eperisone (Epe) once at day 10. After 24 h, whole blood was collected from the mice. Analysis of the blood samples was performed by TRANS GENIC INC. Values represent the mean ± SEM.

**Table 2:** Effects of eperisone administration on gastric and colonic mucosa.

	Vehicle (*n* = 4)	BLM (*n* = 4)	BLM + Epe (250) (*n* = 4)
Diarrhea	n.d.	n.d.	n.d.
Hemorrhagic stool	n.d.	n.d.	n.d.
Gastric mucosal injury	n.d.	n.d.	n.d.
Colonic mucosal injury	n.d.	n.d.	n.d.

Mice were treated with bleomycin (BLM, 1 mg/kg) or vehicle once only on day 0. The mice were then orally administered 250 mg/kg of eperisone (Epe) once at day 10. After 24 h, the fecal condition (diarrhea or hemorrhagic stool) of the mice was visually examined. The analysis of fecal condition was conducted by an investigator blinded to the study protocol. Gastric mucosal injury and colonic mucosal injury were analyzed based on the hematotoxin and eosin staining images shown in Fig. 5.

## Discussion

In this study, we found that eperisone, a central muscle relaxant, preferentially reduces the percentage of viable fibroblasts, an effect not produced by the existing drugs pirfenidone and nintedanib. Moreover, eperisone also inhibited fibroblast activation in vivo and markedly reduced BLM-dependent exacerbation of pulmonary fibrosis. Furthermore, no adverse effects were observed, even when eperisone was administered to mice at a dose five times higher than the dose at which it inhibited BLM-induced pulmonary fibrosis. To the best of our knowledge, this is the first study of the effects of eperisone on fibroblasts and its therapeutic effects on a BLM-induced pulmonary fibrosis model. Eperisone is used clinically to improve muscle tone in patients with lumbago and spastic paralysis caused by cerebrovascular disease, and the maximum daily dose used in Japan is 150 mg (orally). Thus, we calculated the human equivalent dose (HED) using the dose used in animals (15 or 50 mg/kg), animal weight (0.04 kg), and human weight (60 kg) according to a previous report [26] and found that the HED was 1.3 or 4.5 mg/kg. Therefore, a person weighing 60 kg would require a dosage of 78–270 mg per day. Thus, eperisone at its current clinical dose (150 mg/day) is expected to be effective against IPF.

To investigate whether central muscle relaxation is involved in the preferential effects of eperisone on fibroblasts, we examined the percentage of viable LL29 or A549 cells when other central muscle relaxants were administered. As shown in Fig. 3, tolperisone, inaperisone, or lanperisone, but not tizanidine, methocarbamol, or baclofen, preferentially reduced the viability of LL29 cells. Thus, we speculate that eperisone exerts its preferential suppression of fibroblasts by a molecular mechanism other than its muscle relaxant effect. In terms of chemical structure, the drugs that showed fibroblast-preferential effects had higher ClogP values, a lipophilic parameter related to membrane permeability (Supplementary Fig. S4). Therefore, a high ClogP value may be necessary for a drug to exert a preferential effect on fibroblasts. In addition, it is interesting to note that the drugs that preferentially reduced the viable percentage of fibroblasts contain isobutyrophenone bound to the nitrogen atom of the heterocyclic ring in the chemical structure. Chemical modification based on this basic structure may lead to the discovery of drugs that preferentially act on fibroblasts.

Nevertheless, the molecular mechanism by which eperisone preferentially reduced the viability of lung fibroblasts could not be elucidated in this study. A recent study suggested that developmentally regulated brain protein (Drebrin), which binds to and increases the stability of actin filaments in neurons, is mainly expressed in myofibroblasts of mouse hearts after myocardial infarction or mouse lungs after BLM administration and promotes the expression of fibrosis-related genes, such as *α-SMA* and *Col1A1* [27]. Another group demonstrated that radiation-induced DNA damage is reduced in IPF fibroblasts and correlates with activation of the transcription factor forkhead box M1 (FoxM1) and the subsequent upregulation of the DNA repair proteins RAD51 and BRCA2 [28]. Moreover, syndecan-2 is reported to attenuate radiation-induced pulmonary fibrosis in mice and inhibit TGF-β1-induced fibroblast-myofibroblast differentiation, migration, and proliferation by down-regulating phosphoinositide 3-kinase/serine/threonine kinase/Rho-associated coiled-coil kinase signaling and blocking serum response factor binding to the α-SMA promoter via CD148 [29]. Furthermore, microRNA-101 has been reported to inhibit WNT5a (Wnt ligand)-dependent lung fibroblast proliferation by inhibiting NFATc2 signaling and TGF-β1-dependent lung fibroblast activation by inhibiting SMAD2/3 signaling [30]. Taken together, these reports suggest that the molecular mechanisms by which eperisone preferentially reduces the percentage of viable lung fibroblasts may involve previously reported factors that regulate fibroblast activation.

Although pirfenidone and nintedanib are currently used in clinical practice to treat IPF, in some cases, these drugs have not shown efficacy and have been reported to induce adverse effects such as elevation of liver damage markers, diarrhea, and indigestion [5, 6]. Thus, in this study, we conducted a “drug-repositioning strategy” to identify safer and more effective drugs for IPF treatment. The in vitro studies shown in Figs. 1 and 2 revealed that eperisone, but not pirfenidone or nintedanib, exhibited a fibroblast-preferential reduction of viable cells. Moreover, the in vivo studies shown in Fig. 3 and Supplementary Fig. S1 indicated that eperisone, but not pirfenidone or nintedanib, inhibited the exacerbation of BLM-induced pulmonary fibrosis. In addition, eperisone did not induce adverse effects such as hepatotoxicity marker elevation or gastrointestinal disorders. Therefore, we suggest that eperisone may be a safer and more effective treatment for IPF than pirfenidone or nintedanib.

After screening drugs that selectively induce fibroblast cell death, we selected eperisone and showed its efficacy in animal models of IPF, which is caused by fibroblast activation. As mentioned above, eperisone has never been reported to preferentially induce cell death in fibroblasts or effectively treat fibrosis models. However, fibrosis is also induced in organs other than the lungs, such as the liver, heart, and kidneys [31]. For example, in the liver, hepatic stellate cells are activated by stimuli such as TGF-β1 and transdifferentiate into myofibroblasts, which promote the production of extracellular matrix components such as collagen and induce liver fibrosis in diseases such as nonalcoholic steatohepatitis [32]. In the kidney, resident fibroblasts, pericytes, bone marrow-derived cells, and endothelial cells transdifferentiate into myofibroblasts and induce kidney fibrosis [33]. Thus, activated myofibroblasts that transdifferentiate from fibroblasts play a role in promoting fibrosis in organs other than the lungs. Therefore, eperisone, which can preferentially inhibit fibroblast activity, may be effective not only in lung fibrosis models but also in fibrosis models of other organs; thus, the results of this study have promising applications for future research.

## Supplementary information

Supple

## Data Availability

The data that support the findings of our study are available from the corresponding author upon reasonable request.
