# Supplementary material for: Therapeutic effects of eperisone on pulmonary fibrosis via preferential suppression of fibroblast activity
Source: Cell Death Discov. 2022 Feb 8;8:52. doi: 10.1038/s41420-022-00851-7 (PMC8824291; doi:10.1038/s41420-022-00851-7)
Supplement: Supplementary file 1 — Supple [file 41420_2022_851_MOESM1_ESM.docx]

**Therapeutic effects of eperisone on pulmonary fibrosis via preferential suppression of fibroblast activity**

**Ken-ichiro Tanaka**^1, *^**, Mikako Shimoda**^1^**, Toshifumi Sugizaki**^1^**, Maki Ikeda**^1^**, Ayaka Takafuji**^1^**, Masahiro Kawahara**^1^**, Naoki Yamakawa**^2^**, and Tohru Mizushima**^3^**.**

^1^Laboratory of Bio-Analytical Chemistry, Research Institute of Pharmaceutical Sciences, Faculty of Pharmacy, Musashino University, 1-1-20 Shinmachi, Nishi-Tokyo 202-8585, Japan; ^2^Shujitsu University School of Pharmacy, Okayama 703-8516, Japan; ^3^ LTT Bio-Pharma Co., Ltd, Shiodome Building 3F, 1-2-20 Kaigan, Minato-ku, Tokyo 105-0022, Japan.

**Correspondence**

Dr. Ken-ichiro Tanaka, Laboratory of Bio-Analytical Chemistry, Research Institute of Pharmaceutical Sciences, Musashino University, 1-1-20 Shinmachi, Nishitokyo-shi, Tokyo 202-8585, Japan. TEL & FAX: 81-42-468-9335, E-mail: [k-tana@musashino-u.ac.jp](mailto:k-tana@musashino-u.ac.jp)

**Running title**: Eperisone and pulmonary fibrosis

**Keywords:** bleomycin, idiopathic pulmonary fibrosis, fibroblast, eperisone, muscle relaxant.

**Materials and methods**

**Chemicals and animals**

Eperisone hydrochloride, tolperisone hydrochloride, isoflurane, Dulbecco's modified Eagle's medium (DMEM), Eagle's minimal essential medium (EMEM), Roswell Park Memorial Institute (RPMI) 1640 medium, L-hydroxyproline, sodium acetate, trichloroacetic acid (TCA), perchloric acid, and formalin neutral buffer solution were obtained from Fujifilm Wako Pure Chemical Corporation (Tokyo, Japan). Tizanidine hydrochloride, baclofen, methocarbamol and pirfenidone were from Tokyo Chemical Industry (Tokyo, Japan), and nintedanib was from Cayman Chemical (Ann Arbor, MI). Inaperizone hydrochloride and lanperizone hydrochloride were synthesized in our laboratory. Details of the synthesis method are described in the Supplementary Materials and Methods. BLM was from Nippon Kayaku (Tokyo, Japan). Chloramine T and 4-dimethylaminobenzaldehyde (DMBA) were obtained from Sigma (St. Louis, MO). Fetal bovine serum (FBS) was purchased from BioWest (Nuaille, France), and Ham's F-12K (Kaighn's modification) medium was from Thermo Fischer Scientific (Waltham, MA). An antibody against α-smooth muscle actin (α-SMA, code: ab5694) was from Abcam (Cambridge, Cambridgeshire), and Alexa Fluor 594 goat anti-rabbit immunoglobulin G (code: A-11012) were from Invitrogen (Carlsbad, CA). Recombinant human TGF-β1 were from R&D Systems (Minneapolis, MN), and mounting medium for immunohistochemical analysis (VECTASHIELD) was purchased from Vector Laboratories (Burlingame, CA). Mayer’s haematoxylin, 1% eosin alcohol solution, primary mordant agent, secondary mordant agent, 0.75% Orange G solution, Masson's staining solution B, 2.5% phosphotungstic acid solution, aniline blue solution, mounting medium for histological examination (malinol) and Weigert’s iron haematoxylin were from MUTO Pure Chemicals (Tokyo, Japan). 4,6-diamidino-2-phenylindole (DAPI) was purchased from Dojindo (Kumamoto, Japan). The RNeasy kit was obtained from Qiagen (Valencia, CA), PrimeScript™ II 1^st^ strand cDNA Synthesis kit was from TAKARA Bio (Ohtsu, Japan), and THUNDERBIRD® SYBR qPCR Mix was from Bio-Rad (Hercules, CA). ICR mice (6-7 weeks old, male) were purchased from Charles River (Yokohama, Japan). The experiments and procedures described here were carried out in accordance with the Guide for the Care and Use of Laboratory Animals as adopted and promulgated by the National Institutes of Health, and were approved by the Animal Care Committee of Musashino University.

**Treatment of mice with BLM, eperisone, and other reagents**

Mice were anesthetized with isoflurane and intratracheally administered BLM (1 mg/kg, once) in sterile saline via a single channel pipette (P200). Ten days after BLM administration, eperisone (15 or 50 mg/kg), tolperisone (15 mg/kg), pirfenidone (200 mg/kg), and nintedanib (30 mg/kg) were administered orally for a total of 9 days from day 10 to day 18. Various analyses were then performed on day 20.

In the adverse effect study, 10 days after BLM administration, 250 mg/kg of eperisone was orally administered once, which was five times the dose that showed efficacy. Twenty-four hours after eperisone administration, the fecal condition of the mice was visually examined. In addition, plasma samples and stomach and colon tissues were collected from the mice. Analysis of the plasma samples was performed by TRANS GENIC INC. (<https://www.transgenic.co.jp/>). In contrast, analysis of hepatic and renal damage markers in plasma after 9 consecutive days of administration of eperisone at the dose that showed efficacy (50 mg/kg) was performed using Dri-Chem slides (Fujifilm Wako Pure Chemicals, Tokyo, Japan), according to the manufacturer’s protocols.

**Measurement of lung mechanics and FVC**

Measurement of lung mechanics and FVC was performed with a computer-controlled small-animal ventilator connected to a negative pressure reservoir (FlexiVent; SCIREQ, Montreal, Canada), as previously described^1^. Mice were anaesthetised with three types of mixed anesthetic agents (0.75 mg/kg medetomidine, 4.0 mg/kg midazolam, and 5.0 mg/kg butorphanol), a tracheotomy was performed, and an 8 mm-long section of metallic tube (outer and inner diameters of 1.27 mm and 0.84 mm, respectively) was inserted into the trachea. Mice were mechanically ventilated at a rate of 150 breaths/min, using a tidal volume of 8.7 ml/kg and a positive end-expiratory pressure of 2–3 cmH_2_O.

Total respiratory system elastance and tissue elastance were measured by snap shot and forced oscillation techniques, respectively. For determination of FVC, lungs were inflated to 30 cmH_2_O over one second and held at this pressure. After 0.2 sec, the pinch valve (connected to the ventilator) was closed, and after 0.3 sec, the shutter valve (connected to the negative pressure reservoir) was opened, exposing the lung to the negative pressure, which was held for 1.5 sec to ensure complete expiration. All data were analysed using FlexiVent software (version 5.3; SCIREQ, Montreal, Canada).

**Cell culture**

A549 cells (human lung epithelial cell line) were cultured in DMEM supplemented with 10% FBS, LL29 cells (lung fibroblasts from an IPF patient) and HFL-1 cells (human fetal-derived normal lung fibroblasts) were cultured in Ham's F-12K (Kaighn's Modification) supplemented with 15% FBS, and IMR-90 cells (human fetal-derived normal lung fibroblasts) were cultured in EMEM supplemented with 10% FBS and non-essential amino acids, respectively, in a humidified atmosphere of 95% air with 5% CO_2_ at 37°C. RL-34 cells (rat liver-derived normal epithelial cells) were cultured in DMEM supplemented with 10% FBS, and RI-T cells (rat hepatic stellate cells) were cultured in RPMI 1640 medium supplemented with 10% FBS, respectively, in a humidified atmosphere of 95% air with 5% CO_2_ at 37°C. A549, LL29, and IMR-90 cells were purchased from the American Type Culture Collection (Manassas, VA), and HFL-1, RL-34, and RI-T cells were purchased from the Japanese Collection of Research Bioresources Cell Bank (Osaka, Japan).

Viable cells were measured as previously described^2, 3^. Briefly, dissociated A549, LL29, HFL-1, IMR-90, RL-34, or RI-T cells were added to 96-well culture plates at a concentration of 1 × 10^4^ cells per well in 200 μl of culture medium. After a 24-h incubation, cells were treated with various reagents added to the medium. After 24 h, the percentage of viable cells was quantified using CellTiter-Glo® 2.0 (Promega Corporation, Madison, WI, USA). The cytotoxicity in LL29 cells after eperisone addition was measured every hour using CellTox™ Green Dye (Promega Corporation, Madison, WI, USA) and a microplate reader (Tecan, Kawasaki, Japan; excitation: 485 nm, emission: 530 nm).

**Real-time RT-PCR analysis**

Total RNA was extracted from LL29 cells or lung tissue using an RNeasy kit (Qiagen, Hilden, Germany) according to the manufacturer’s protocol. Using a PrimeScript™ II 1^st^ strand cDNA Synthesis kit, samples were reverse-transcribed and THUNDERBIRD® SYBR qPCR Mix, Bio-Rad’s CFX96™ Real-time system, and CFX Manager™ software (Hercules, CA) were used for real-time RT-PCR experiments. Electrophoretic analysis of reaction products was done to confirm specificity. Glyceraldehyde-3-phosphate dehydrogenase (GAPDH) or hypoxanthine phosphoribosyltransferase 1 (HPRT1) cDNA was used as an internal standard. Primers were designed using either Primer3 or Primer-BLAST. Primer sequences will be provided upon request.

**Histological and immunohistochemical analyses**

Tissue samples were fixed in 10% formalin neutral buffer solution for 24 h, and then embedded in paraffin before being cut into 4 µm-thick sections.

For staining of collagen (Masson’s trichrome staining), sections were treated sequentially with primary mordant agent, Weigert’s iron haematoxylin, secondary mordant agent, 0.75% Orange G solution, Masson's staining solution B, 2.5% phosphotungstic acid solution, and finally with aniline blue solution. Samples were mounted with malinol, and inspected with a fluorescence microscope (Olympus DP71) or scanned using a NanoZoomer-XR digital slide scanner. Image J software (National Institutes of Health, Bethesda, MD) was used to calculate the percentage of collagen positive area.

For immunohistochemical analysis of α-SMA, sections were blocked with 2.5% goat serum for 10 min, incubated for 12 h with an antibody against α-SMA (1:100 dilution) in the presence of 2.5% bovine serum albumin, and then incubated with Alexa Fluor 594 goat anti-rabbit immunoglobulin G (1:500 dilution) and DAPI (5 µg/ml) for 1 h. Samples were mounted with VECTASHIELD and inspected with a fluorescence microscope (Olympus DP71). Image J software (National Institutes of Health, Bethesda, MD) was used to calculate the percentage of α-SMA positive area.

For histological examination, sections were stained first with Mayer’s haematoxylin and then with 1% eosin alcohol solution (H&E staining). Samples were mounted with malinol and inspected with a fluorescence microscope (Olympus DP71).

**Hydroxyproline determination**

Hydroxyproline content was determined as previously described^4^. Briefly, the lung was removed and homogenised in 1.0 ml of 5% trichloroacetic acid. After centrifugation, pellets were hydrolysed in 0.5 ml of 10 N HCl for 16 h at 110°C. Each sample was incubated for 20 min at room temperature with 0.5 ml of 1.4% w/v chloramine T solution, and then incubated at 65°C for 10 min with 0.5 ml of Ehrlich’s reagent (1 M DMBA, 70% v/v isopropanol and 30% v/v perchloric acid). The absorbance of each sample was then measured at 550 nm to determine the amount of hydroxyproline present.

**Reagents used for chemical synthesis**

All used reagents and solvents for organic syntheses were purchased from Tokyo Chemical Industry Co., Ltd (Tokyo, Japan) or Wako Pure Chemical Industries (Tokyo, Japan) and used without further purification. Melting point (mp) data were recorded on a Yanaco MP-J3 micro-melting point apparatus (Yanako Equipment Research and Development Laboratory, Kyoto, Japan). ^1^H-NMR (500 MHz) spectra and ^13^C-NMR (125 MHz) spectra were measured on a JEOL ECA-500 spectrometer (JEOL, Tokyo, Japan). High-resolution mass spectra (HRMS) were detected with an ESI-TOF mass spectrometer (Bruker MicroTOF, Bruker, Bremen, Germany) in the positive mode.

**Inaperison hydrochloride**

The mixture of 4’-ethylpropiophenone (1.02 mL, 6.2 mmol) and pyrrolidine hydrochloride (0.81 g, 7.6 mmol) in 1,3-dioxolane (1.7 ml) was stirred for 8 h at 90˚C. After cooling to room temperature, the reaction mixture was diluted with H_2_O, and organic layer was extracted with ethyl acetate. The extracted solution was evaporated to dryness, and the resulting residue was treated with hydrochloric acid in acetone-ether to give white solid (mp: 184-186˚C) in 80% yield (1.4 g). This compound is known, but commercially unavailable compound for which the chemical structure could be identified from the ^1^H- and ^13^C-NMR spectra; ^1^H-NMR (D_2_O): *δ* = 7.79 (d, 2H, *J*=8.0 Hz), 7.25 (d, 2H, *J*=8.0 Hz), 4.66 (s, 2H), 3.88-3.95 (m, 1H), 3.57 (dd, 1H, *J*=9.2, 9.6 Hz), 3.14 (dd, 2H, *J*=7.5, 13.2 Hz), 2.98-3.46 (br, 1H), 2.53 (q, 2H, *J*=7.4 Hz), 1.88 (brs, 4H), 1.09 (d, 3H, *J*=6.9 Hz), 1.03 (t, 3H, *J*=7.4 Hz); ^13^C-NMR (D_2_O): *δ* = 203.4, 152.6, 131.9, 129.0, 128.7, 56.2, 54.9, 38.2, 28.5, 22.6, 16.6, 14.5; HRMS calcd. for C_16_H_24_NO [M+H]^+^: 246.1858; found: m/z = 246.1856.

**Lanperison hydrochloride (racemate)**

Synthesis of inaperison hydrochloride is followed, except that 1.25 g (6.2 mmol) of 4’-(trifluoromethyl)propiophenone are used as starting substance instead of 4’-ethylpropiophenone to obtain the title compound in a yield of 1.6g (80%, white solid), mp: 176-178˚C. This compound is known, but commercially unavailable compound for which the chemical structure could be identified from the ^1^H- and ^13^C-NMR spectra; ^1^H-NMR (D_2_O): *δ* = 7.99 (d, 2H, *J*=8.0 Hz), 7.71 (d, 2H, *J*=8.0 Hz), 4.66 (s, 2H), 3.94-4.01 (m, 1H), 3.63 (dd, 1H, *J*=8.6, 12.6 Hz), 3.19 (dd, 1H, *J*=4.6, 12.6), 3.02-3.48 (br, 2H), 1.91 (brs, 4H), 1.13 (d, 3H, *J*=7.4 Hz); ^13^C-NMR (D_2_O): *δ* = 202.8, 137.3, 134.5 (q, *J*_C-F_=32 Hz), 129.2, 126.1, 124.7, 122.5, 56.0, 38.7, 22.6, 16.1; HRMS calcd. for C_15_H_19_F_3_NO [M+H]^+^: 286.1419; found: m/z = 286.1421.

|  | **vehicle (n = 6)** | **BLM (n = 7)** | **BLM + Epe (50) (n = 7)** |
| --- | --- | --- | --- |
| **AST (U/L)** | 36.3 ± 2.9 | 44.4 ± 2.0 | 52.7 ± 4.6 |
| **ALT (U/L)** | 26.0 ± 2.5 | 24.6 ± 3.0 | 30.7 ± 4.7 |
| **BUN (mg/dL)** | 23.6 ± 2.3 | 18.2 ± 1.5 | 19.1 ± 2.0 |
| **Creatinine (mg/dL)** | 0.39 ± 0.06 | 0.25 ± 0.03 | 0.27 ± 0.02 |

**Supplementary Table S1. Effect of eperisone administration on biochemical markers in the plasma.**

Mice were treated with bleomycin (BLM, 1 mg/kg) or vehicle once only on day 0. The mice were then orally administered eperisone (Epe, 50 mg/kg) once daily for 9 days (from day 10 to day 18). Plasma samples were collected from the mice on day 20, and their urea AST, ALT, BUN and creatinine concentrations were determined using Dri-Chem slides.

**
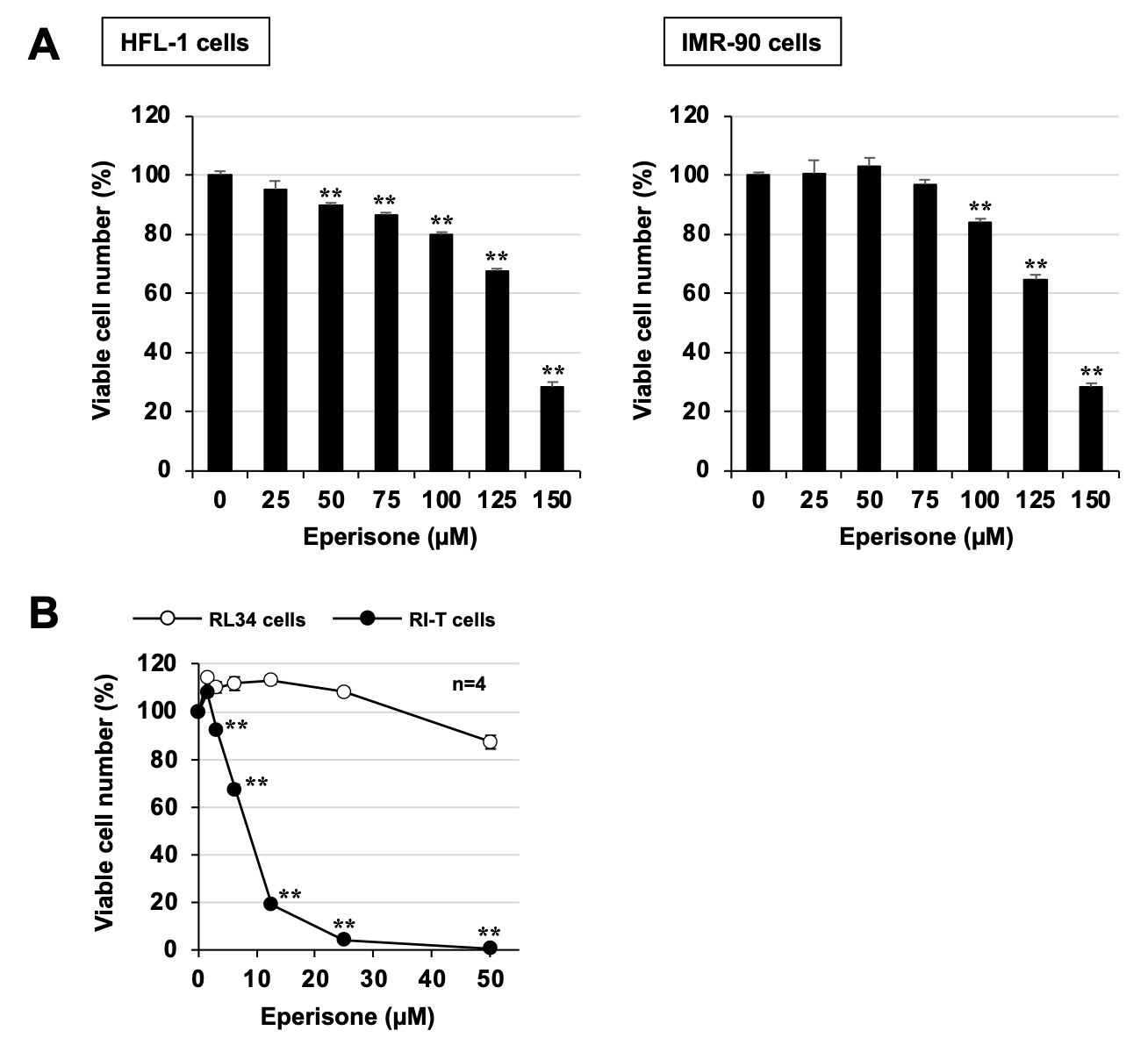
**

**Supplementary Figure S1. Effect of eperisone on the number of viable cells in other cells.**

HFL-1, IMR-90, RL-34, or RI-T cells were incubated with the indicated concentrations (µM) of eperisone for 24 h. The percentage of viable cells was determined using a CellTiter-Glo® 2.0 assay (A, B). Values represent the mean ± SEM **P < 0.01; *P < 0.05; NS, not significant.


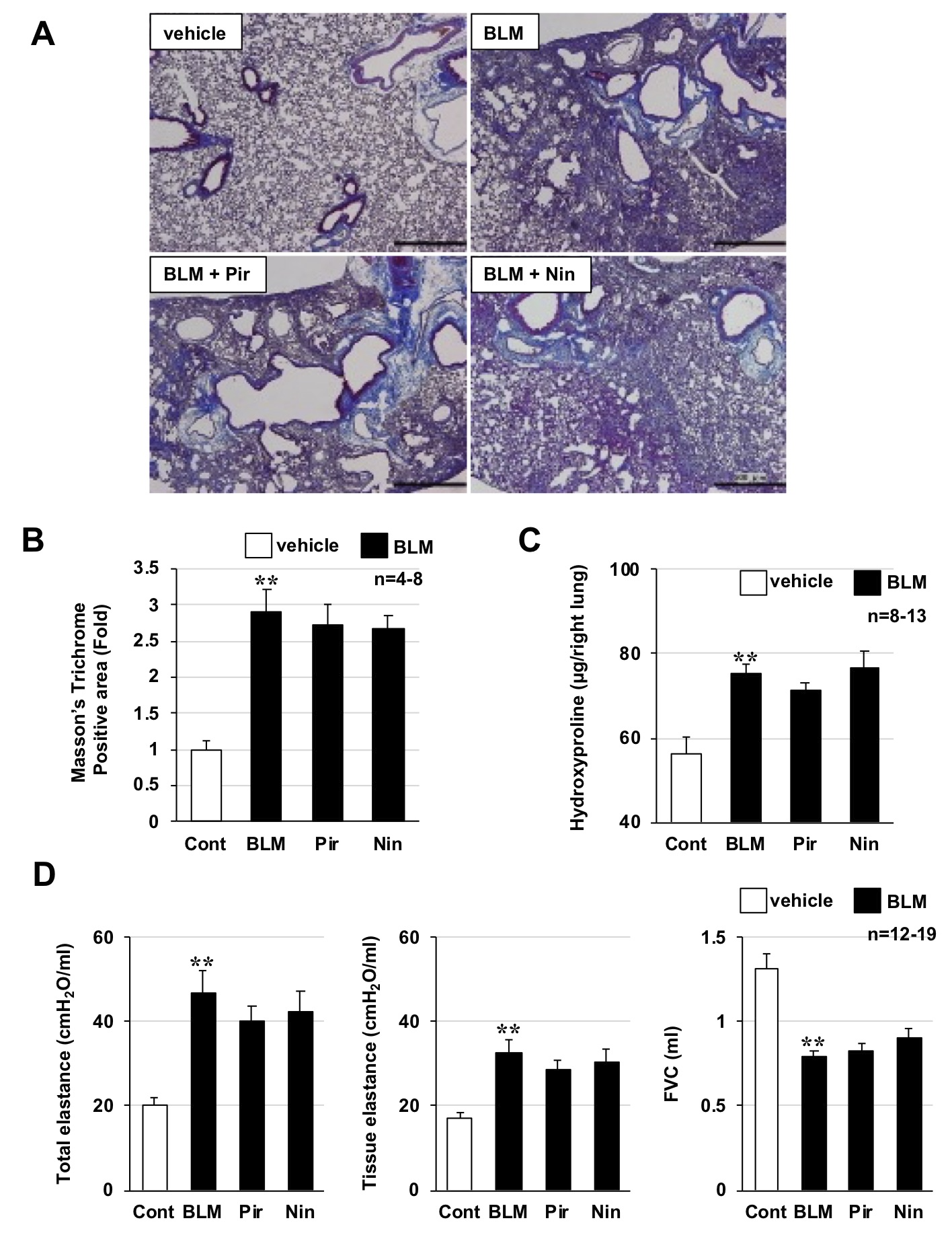


**Supplementary Figure S2. Effect of other anti-fibrotic drugs on pre-developed pulmonary fibrosis.**

Mice were treated with bleomycin (BLM, 1 mg/kg) or vehicle once only on day 0. The mice were then orally administered pirfenidone (Pir, 200 mg/kg) or nintedanib (Nin, 30 mg/kg) once daily for 9 days (from day 10 to day 18). Pulmonary tissue sections were prepared on day 20 and subjected to histopathological examination (Masson’s trichrome staining; scale bar = 500 µm) (A). The collagen-positive area was determined based on Masson’s trichrome staining images (B). The pulmonary hydroxyproline level was determined on day 20 (C). The total respiratory system elastance, tissue elastance, and forced vital capacity (FVC) were measured on day 20 (D). Values represent the mean ± SEM ***P < 0.01*; **P < 0.05*; NS, not significant.


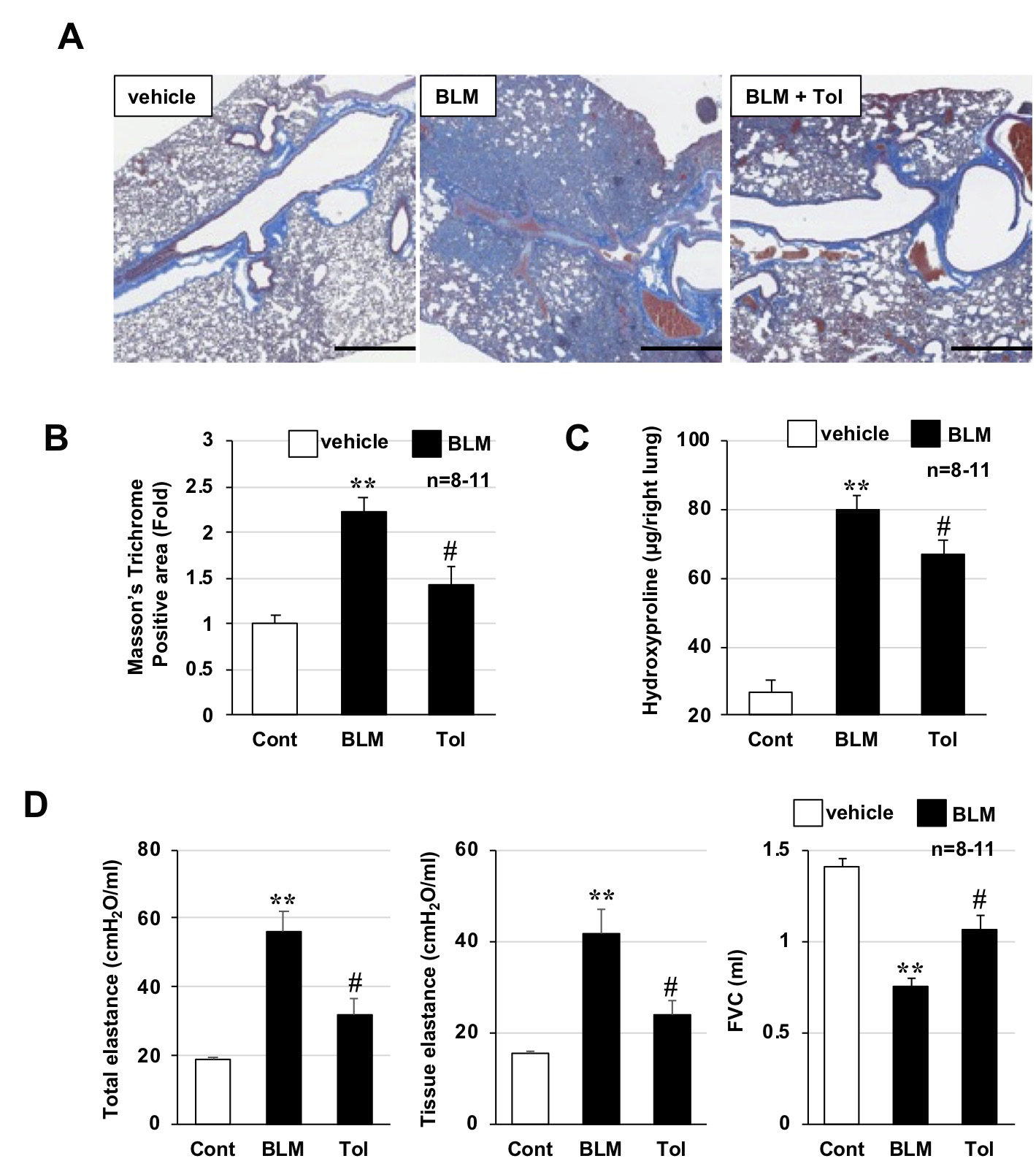


**Supplementary Figure S3. Effect of tolperisone on pre-developed pulmonary fibrosis.**

Mice were treated with bleomycin (BLM, 1 mg/kg) or vehicle once only on day 0. The mice were then orally administered tolperisone (Tol, 15 mg/kg) once daily for 9 days (from day 10 to day 18). Pulmonary tissue sections were prepared on day 20 and subjected to histopathological examination (Masson’s trichrome staining; scale bar = 500 µm) (A). The collagen-positive area was determined based on Masson’s trichrome staining images (B). The pulmonary hydroxyproline level was determined on day 20 (C). The total respiratory system elastance, tissue elastance, and forced vital capacity (FVC) were measured on day 20 (D). Values represent the mean ± SEM ***P < 0.01*; **P < 0.05*; NS, not significant.


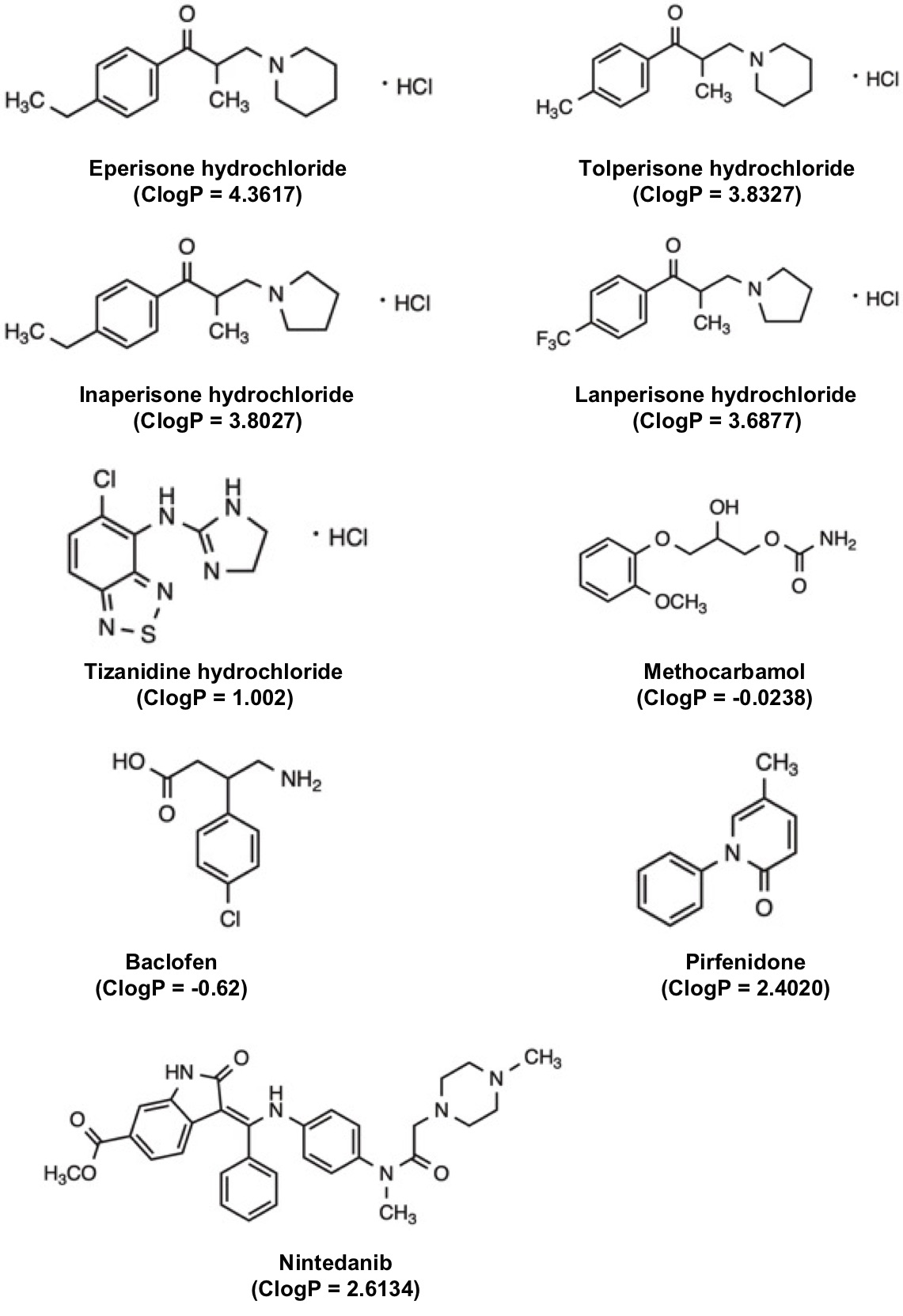


**Supplementary Figure S4. Chemical structures of the drugs used in this study.**

The chemical structures and ClogP values of the drugs used in this study are shown. The ClogP values were calculated using ChemDraw v19.1 software.

**References**

1. Tanaka K, Azuma A, Miyazaki Y, Sato K, Mizushima T. Effects of lecithinized superoxide dismutase and/or pirfenidone against bleomycin-induced pulmonary fibrosis. *Chest* 2012, **142**(4)**:** 1011-1019.

2. Nakano Y, Shimoda M, Okudomi S, Kawaraya S, Kawahara M, Tanaka KI. Seleno-l-methionine suppresses copper-enhanced zinc-induced neuronal cell death via induction of glutathione peroxidase. *Metallomics* 2020, **12**(11)**:** 1693-1701.

3. Tanaka KI, Shimoda M, Kasai M, Ikeda M, Ishima Y, Kawahara M. Involvement of SAPK/JNK Signaling Pathway in Copper Enhanced Zinc-Induced Neuronal Cell Death. *Toxicol Sci* 2019, **169**(1)**:** 293-302.

4. Woessner JF, Jr. The determination of hydroxyproline in tissue and protein samples containing small proportions of this imino acid. *Arch Biochem Biophys* 1961, **93:** 440-447.
